# Oxidation Stress as a Mechanism of Aging in Human Erythrocytes: Protective Effect of Quercetin

**DOI:** 10.3390/ijms23147781

**Published:** 2022-07-14

**Authors:** Alessia Remigante, Sara Spinelli, Nancy Basile, Daniele Caruso, Giuseppe Falliti, Silvia Dossena, Angela Marino, Rossana Morabito

**Affiliations:** 1 Department of Chemical, Biological, Pharmaceutical and Environmental Sciences, University of Messina, 98122 Messina, Italy; aremigante@unime.it (A.R.); sspinelli@unime.it (S.S.); nebasile95@gmail.com (N.B.); marinoa@unime.it (A.M.); 2 Complex Operational Unit of Clinical Pathology, Papardo Hospital, 98158 Messina, Italy; caruso.daniele1985@libero.it (D.C.); peppefal@tin.it (G.F.); 3 Institute of Pharmacology and Toxicology, Paracelsus Medical University, 5020 Salzburg, Austria; silvia.dossena@pmu.ac.at

**Keywords:** quercetin, d-Galactose, aging, band 3 protein function, oxidative stress, glycation

## Abstract

Aging is a multi-factorial process developing through a complex net of interactions between biological and cellular mechanisms and it involves oxidative stress (OS) as well as protein glycation. The aim of the present work was to verify the protective role of Quercetin (Q), a polyphenolic flavonoid compound, in a d-Galactose (d-Gal)-induced model of aging in human erythrocytes. The anion-exchange capability through the Band 3 protein (B3p) measured by the rate constant of the SO_4_^2−^ uptake, thiobarbituric acid reactive substances (TBARS) levels—a marker of lipid peroxidation—total sulfhydryl (-SH) groups, glycated hemoglobin (A1c), and a reduced glutathione/oxidized glutathione (GSH-GSSG) ratio were determined following the exposure of erythrocytes to 100 mM d-Gal for 24 h, with or without pre-incubation with 10 µM Q. The results confirmed that d-Gal activated OS pathways in human erythrocytes, affecting both membrane lipids and proteins, as denoted by increased TBARS levels and decreased total sulfhydryl groups, respectively. In addition, d-Gal led to an acceleration of the rate constant of the SO_4_^2^^−^ uptake through the B3p. Both the alteration of the B3p function and oxidative damage have been improved by pre-treatment with Q, which preferentially ameliorated lipid peroxidation rather than protein oxidation. Moreover, Q prevented glycated A1c formation, while no protective effect on the endogenous antioxidant system (GSH-GSSG) was observed. These findings suggest that the B3p could be a novel potential target of antioxidant treatments to counteract aging-related disturbances. Further studies are needed to confirm the possible role of Q in pharmacological strategies against aging.

## 1. Introduction

**Figure 1:**
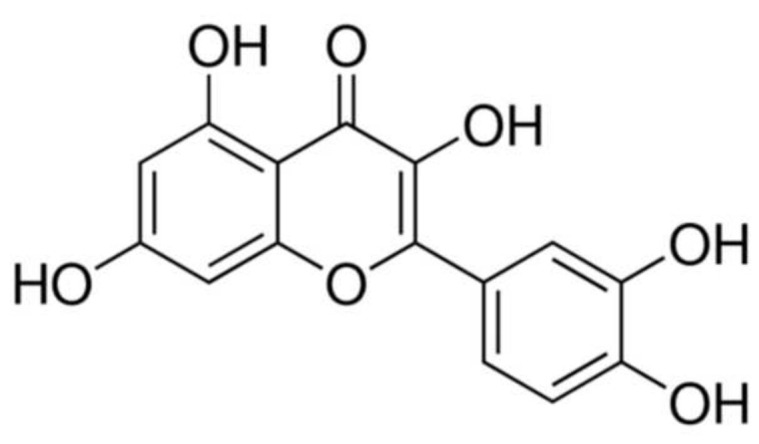
Chemical structure of Quercetin.

Flavonoids are a class of naturally occurring polyphenolic compounds and are important antioxidants and free radical scavengers characterized by high biochemical activity and a variety of pharmacological effects [1,2,3,4,5]. These natural compounds counteract the formation of reactive oxygen species (ROS), which are normally produced as by-products of the cellular metabolism or induced by extracellular stimuli [6,7]. ROS play an important role in cell homeostasis and normal cell functioning. However, their overproduction and accumulation, known as oxidative stress (OS), alters the balance between cellular oxidants and antioxidants [1,8,9,10]. The basic chemical structure of flavonoids consists of two phenyl rings (A and B) and a heterocyclic C ring. To exert their radical-scavenging activity and/or antioxidant potential, flavonoids have three structural groups: (a) the o-dihydroxy (catechol) structure of the B ring, (b) the 2,3-double bond in conjugation with a 4-oxo-function, and (c) the presence of 7, 5, and 3-hydroxyl groups. Quercetin (3,3,4,5,7-pentahydroxyflavone; Q) (Figure 1), one of the flavonoids most widely distributed in plants, displays these three structural requirements [11]. The antioxidant potential of Q has been demonstrated in a wide range of in vitro and in vivo conditions [12,13,14,15,16,17]. This evidence highlights that the antioxidant effect of Q affords protection of the brain, heart, and other tissues against ischemia–reperfusion injury, toxic compounds, and other factors that can induce OS [18]. Additional studies showed that plant polyphenols could be promising agents for pharmacological interventions against specific age-associated diseases through a variety of beneficial biological mechanisms [2,3,4,5].

Aging is a dynamic and progressive functional decline characterized by the accumulation of biological changes that lead to several abnormalities in living systems [19,20,21,22]. In this context, Q appears to be active in different diseases related to aging, such as cancer, diabetes, and cardiovascular and neurodegenerative pathologies [23,24,25,26]. An aging model based on d-galactose (d-Gal) application is widely used in the study of the pharmacodynamics of anti-aging compounds [27] due to its convenience, least side effects, and the high survival rate of experimental animals and cells throughout the experimental period [20,28]. In this regard, Remigante and collaborators proposed a model of early aging in human erythrocytes represented by red blood cells incubated with high doses of d-Gal [29]. The most obvious effects of aging on erythrocytes are, firstly, the increase in membrane lipid peroxidation and the reduction in the intracellular content of glutathione (GSH), and secondly, the glycation of hemoglobin, which is closely linked to Band 3 protein (B3p) function [29]. In addition, other authors have also verified changes in the link between ankyrin and glyceraldehyde 3-phosphate dehydrogenase (GAPDH) [30,31].

Erythrocytes aging impairs the cellular ability to neutralize ROS [32], thus enhancing the susceptibility to oxidative damage. The consequences of the OS in this process include: (a) distorted calcium homeostasis; (b) caspase-3 activation; (c) denatured oxidized Hb (hemichromes) formation; (d) exposure of the neo-antigens on the B3p, and (e) B3p clustering. Hence, a synergic action according to which the above-described oxidative processes increase in older cells, further exacerbating the aging process, is established [10,33]. Alternatively, natural aging can initiate non-enzymatic glycation reactions with free amino acid groups to form advanced glycation end-products (AGEs) [34]. In erythrocytes, the accumulation of AGEs induces structural changes of the proteins, thus leading to the modification of their biochemical functions [35] and increasing OS levels [36,37]. In this regard, Quercetin has been identified as an efficient anti-glycation agent that inhibits the formation of the advanced glycation end-products (AGEs) in several cell types, including erythrocytes [38,39,40].

One of the targets affected by aging-related modifications is the B3p. The Band 3 protein is a Cl^−^/HCO_3_^−^ exchanger with several functions; indeed, it is responsible for gas exchange, ionic equilibrium through the cell membrane, and control of the flexibility, shape, and osmotic properties of the erythrocyte [41]. In addition, it interacts with several enzymes of the glycolytic pathway and establishes contacts with the cytoskeleton and hemoglobin [42,43,44]. Its function can be monitored by the rate constant for the sulphate (SO_4_^2−^) uptake [43,45,46], which is slower and more easily detectable than the Cl^−^ or HCO_3_^−^ uptake [47,48,49,50]. The SO_4_^2−^ uptake measurement has been previously confirmed as a suitable tool to verify the impact of redox conditions on erythrocytes homeostasis [8,43,49,51].

Based on these considerations, in the present study, we investigated the possible protective effect of Q (10 µM) on a model of early aging represented by human erythrocytes treated with 100 mM d-Gal. As it is widely demonstrated that aging is associated with increased OS as well as glycation events [34], both processes were investigated.

## 2. Results

### 2.1. Thiobarbituric Acid Reactive Substances (TBARS) Levels: Effective Concentration of Q

**Figure 2:**
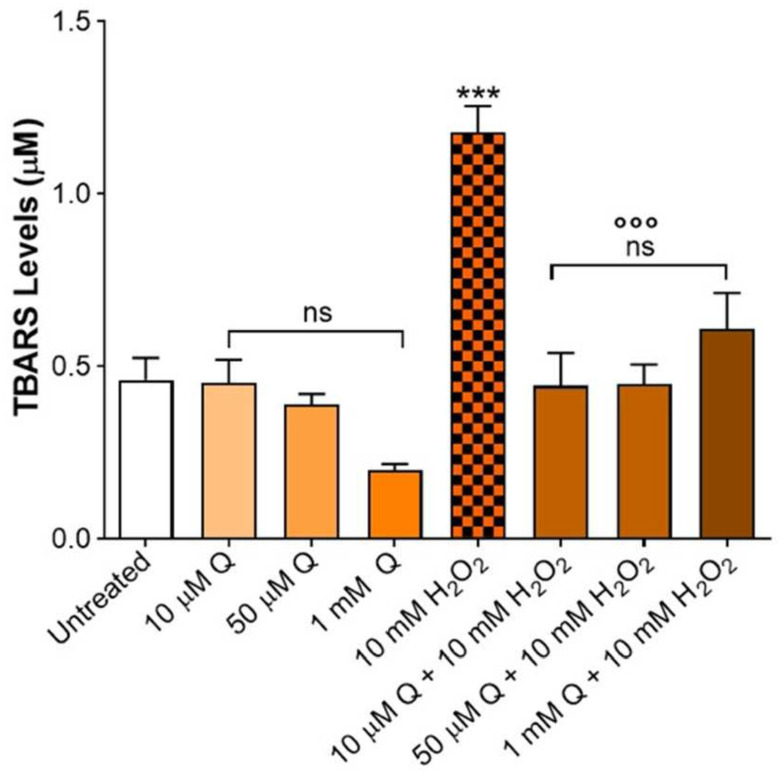
TBARS levels measured in erythrocytes left untreated or after treatment with increasing concentrations (10 µM, 50 µM, or 1 mM) of Q or 10 mM H_2_O_2_ (positive control) with or without pre-incubation with Q. ns, not statistically significant versus untreated (control); ***, *p* < 0.001 versus control; ^°°°^, *p* < 0.001 versus 10 mM H_2_O_2_, one-way ANOVA followed by Bonferroni’s post hoc test (*n* = 10).

The treatment of the erythrocytes with a strong oxidizing agent (10 mM H_2_O_2_ for 1 h, positive control) induced a significant increase in the thiobarbituric acid reactive substances (TBARS) levels compared to the untreated cells (control). Conversely, treatment with 10 µM, 50 µM, or 1 mM Q for 1 h did not significantly affect the (TBARS) levels compared to the control (Figure 2). In the erythrocytes pre-exposed to either 10 µM, 50 µM, or 1 mM Q, and then exposed to 10 mM H_2_O_2_, the TBARS levels were significantly lower (*p* < 0.001) than those observed after exposure to 10 mM H_2_O_2_, while remaining unchanged with respect to the control. Based on these results, the lowest effective concentration of Q (10 µM), which displays a strong antioxidant effect, has been used for further analysis.

### 2.2. SO_4_^2−^ Uptake Measurement

**Figure 3:**
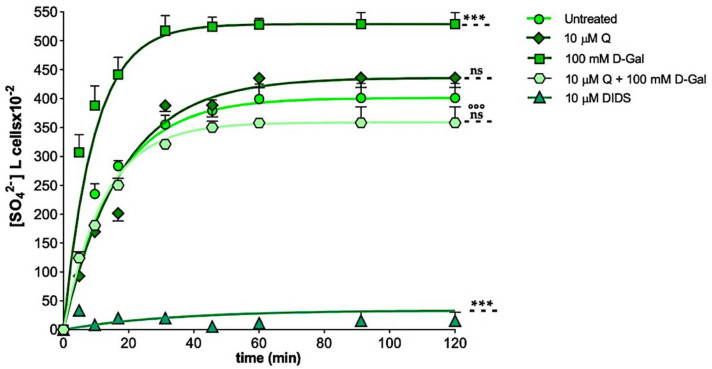
Time course of SO_4_^2−^ uptake in erythrocytes left untreated (control) or treated with 10 µM Q, 100 mM d-Gal with or without pre-exposure to 10 µM Q, or 10 µM DIDS. ns, not statistically significant versus control; ***, *p* < 0.001 versus control; ^°°°^, *p* < 0.001 versus 100 mM d-Gal-treated erythrocytes, one-way ANOVA followed by Bonferroni’s post hoc test.

Figure 3 describes the SO_4_^2−^ uptake as a function of time in the untreated (control) erythrocytes and in the erythrocytes treated with 10 µM Q for 1 h or 100 mM d-Gal for 24 h with or without pre-incubation with 10 µM Q for 1 h at 37 °C. In the control conditions, the SO_4_^2−^ uptake progressively increased and reached equilibrium within 45 min (rate constant of SO_4_^2−^ uptake = 0.064 ± 0.001 min^−1^). The erythrocytes treated with 10 µM Q showed a rate constant of the SO_4_^2−^ uptake that was not significantly different with respect to the control (0.063 ± 0.002 min^−1^). On the contrary, the rate constant value in the erythrocytes treated with 100 mM d-Gal (0.106 ± 0.001 min^−1^) was significantly increased with respect to the control (***, *p* < 0.001). In the erythrocytes pre-incubated with 10 µM Q and then exposed to 100 mM d-Gal, the rate constant (0.080 ± 0.001 min^−1^) was significantly lower than that of the erythrocytes treated with 100 mM d-Gal, but it was not significantly different with respect to the control (Table 1). The SO_4_^2−^ uptake was almost completely blocked by the 10 µM DIDS applied at the beginning of the incubation in the SO_4_^2−^ medium (0.016 ± 0.001 min^−1^, ***, *p* < 0.001, Table 1).

Additionally, the SO_4_^2−^ amount internalized by the 100 mM d-Gal-treated erythrocytes after 45 min of incubation in the SO_4_^2−^ medium was significantly higher compared to the control (***, *p* < 0.001, Table 1). On the contrary, the SO_4_^2−^ amount internalized by the erythrocytes pre-incubated with 10 µM Q and then exposed to 100 mM d-Gal was significantly lower compared to the 100 mM d-Gal-treated erythrocytes (^$$$^, *p* < 0.001, Table 1). In the DIDS-treated cells, the SO_4_^2−^ amount internalized (5.49 ± 3.50) was significantly lower than that determined in both the control or the d-Gal-treated erythrocytes (***, *p* < 0.001, Table 1).

### 2.3. Thiobarbituric Acid Reactive Substances (TBARS) Levels

**Figure 4:**
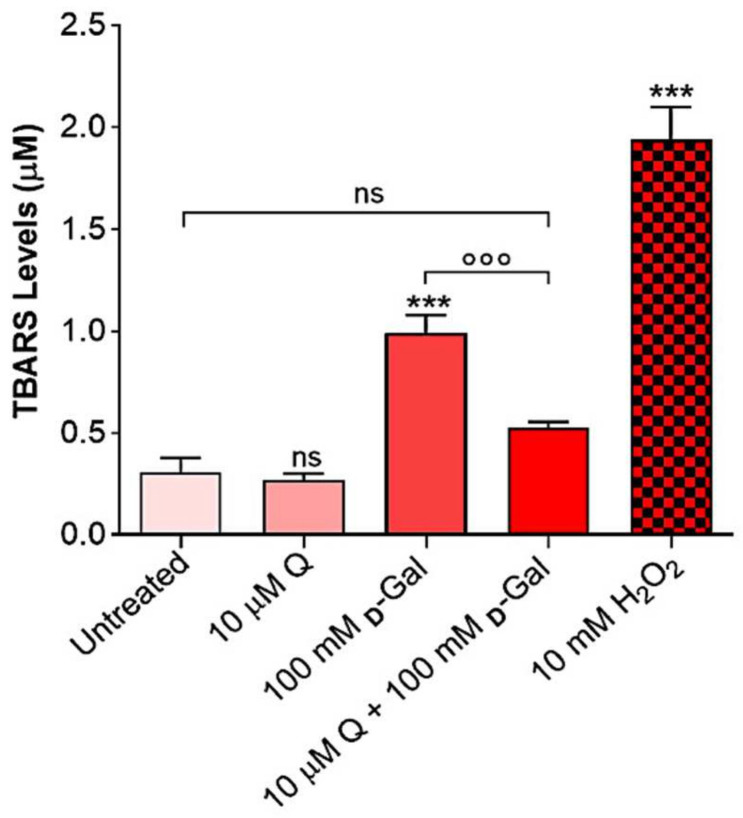
TBARS levels (µM) in untreated erythrocytes (control) and in erythrocytes treated for 24 h with 100 mM **d**-Gal, or, alternatively, with 10 µM Q (pre-incubation for 1 h) with or without **d**-Gal. ns, not statistically significant versus untreated; ***, *p* < 0.001 significant versus control; ^°°°^, *p* < 0.001 versus 100 mM d-Gal, one-way ANOVA followed by Bonferroni’s post hoc test (*n* = 5).

The thiobarbituric acid reactive substances (TBARS) measurements in the erythrocytes are reported in Figure 4. As expected, the TBARS levels of the erythrocytes treated with 10 mM H_2_O_2_ for 30 min were significantly higher with respect to those of the untreated erythrocytes. Similarly, after a 24 h incubation with 100 mM d-Gal, the TBARS levels were significantly increased with respect to those of the untreated erythrocytes. Importantly, in the erythrocytes pre-treated with 10 µM Q and then exposed to 100 mM d-Gal, the TBARS levels were significantly reduced compared to those measured in the 100 mM d-Gal-treated erythrocytes. Q alone (10 µM) did not significantly affect the TBARS levels.

### 2.4. Total Sulfhydryl Group Content Measurement

**Figure 5:**
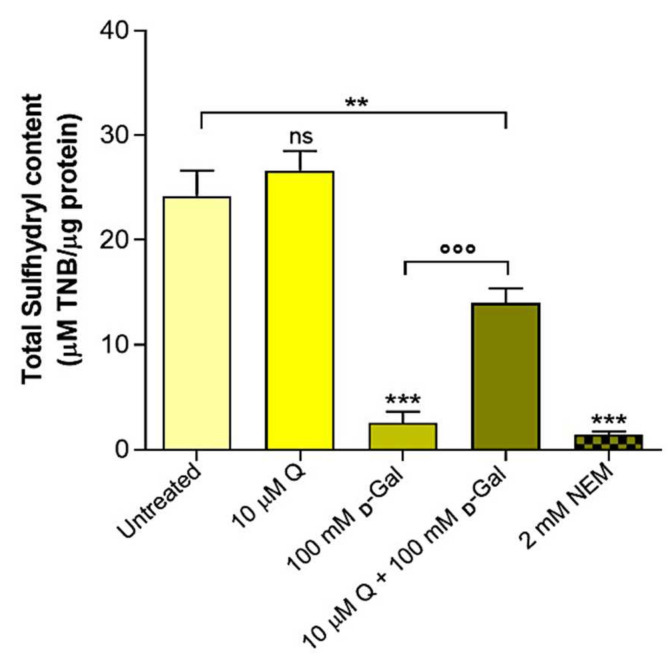
Sulfhydryl group content (µM TNB/µg protein) in untreated erythrocytes (control) and in erythrocytes treated with 100 mM d-Gal for 24 h with or without 10 µM Q (pre-incubation for 1 h), or alternatively, with 2 mM NEM or 10 µM Q for 1 h. ns, not statistically significant versus control; **, *p* < 0.01 and ***, *p* < 0.001 versus control; ^°°°^, *p* < 0.001 versus 100 mM d-Gal, one-way ANOVA followed by Bonferroni’s multiple comparison post hoc test (*n* = 6).

Figure 5 shows the total sulfhydryl group content (µM TNB/µg protein) of the erythrocytes left untreated or treated with either the oxidizing compound NEM (2 mM for 1 h as the positive control), 10 µM Q for 1 h, or 100 mM d-Gal for 24 h with or without pre-treatment with 10 µM Q. As expected, the exposure to the NEM led to a significant reduction in the sulfhydryl group content. The sulfhydryl groups in the 100 mM d-Gal-treated erythrocytes were also significantly reduced with respect to the control (untreated erythrocytes). Pre-treatment with 10 µM Q significantly restored the total sulfhydryl group content in the 100 mM d-Gal-treated erythrocytes. Q alone (10 µM) did not significantly affect the total sulfhydryl group content.

### 2.5. Glycated Hemoglobin Levels

**Figure 6:**
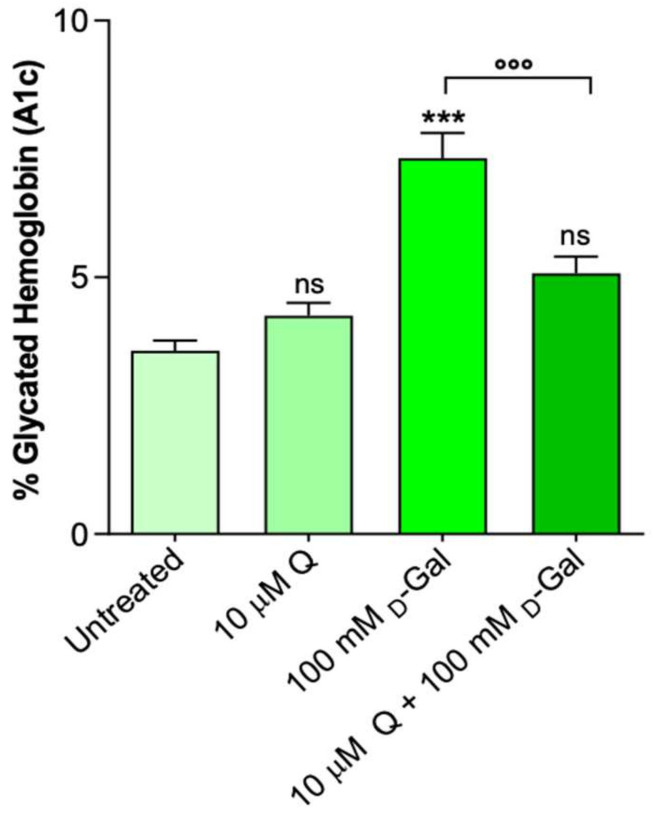
Glycated hemoglobin content (%A1c) in erythrocytes left untreated or incubated for 24 h with 100 mM d-Gal with or without pre-exposure to 10 µM Q, or 10 µM Q alone. ns, not statistically significant versus untreated; ***, *p* < 0.001 versus untreated; ^°°°^, *p* < 0.001 versus 100 mM d-Gal, one-way ANOVA with Bonferroni’s multiple comparison post hoc test (*n* = 8).

Figure 6 shows the glycated hemoglobin levels (%A1c) measured in the erythrocytes left untreated or treated with 10 µM Q for 1 h or 100 mM d-Gal for 24 h with or without pre-treatment with 10 µM Q. The %A1c levels measured following the exposure to d-Gal were significantly increased with respect to those of the control (untreated erythrocytes). Pre-incubation with 10 µM Q for 1 h significantly reduced the %A1c levels in the 100 mM d-Gal-treated erythrocytes toward values that did not differ from the control values. Q alone (10 µM) did not significantly affect the %A1c content.

### 2.6. GSH/GSSG Ratio Measurement

**Figure 7:**
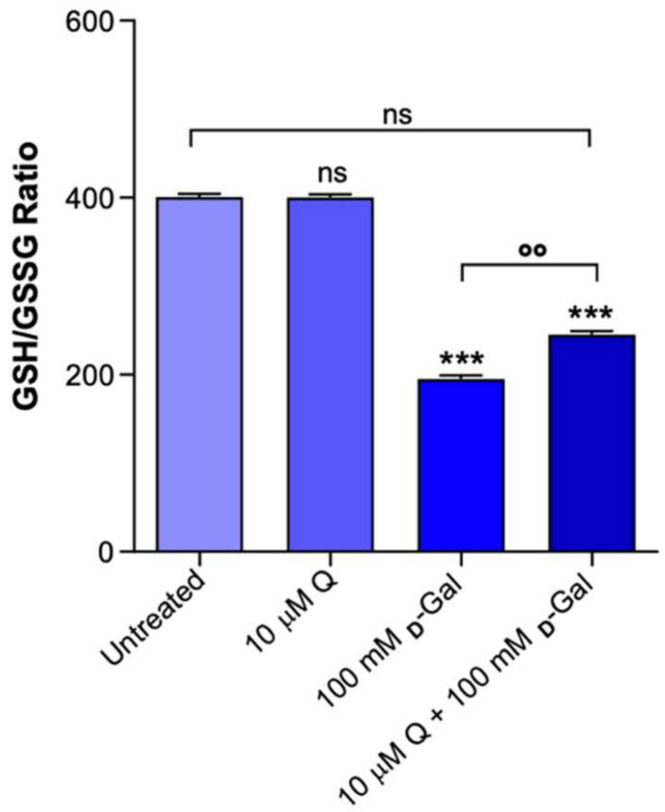
Intracellular GSH/GSSG ratio measured in erythrocytes left untreated or incubated for 24 h with 100 mM d-Gal with or without pre-treatment with 10 µM Q or 10 µM Q alone. ns, not statistically significant versus untreated; ***, *p* < 0.001 versus untreated (control); ^°°^, *p* < 0.01 versus 100 mM d-Gal, one-way ANOVA followed by Bonferroni’s multiple comparison post hoc test (*n* = 7).

Figure 7 shows the GSH/GSSG ratio measured in the erythrocytes treated with d-Gal and/or Q or left untreated. As expected, the GSH/GSSG ratio measured after 24 h of incubation with d-Gal (100 mM) was significantly lower (*p* < 0.001) than that detected in the untreated erythrocytes (control). In parallel, the GSH/GSSG ratio in the erythrocytes pre-incubated with 10 µM Q and then exposed to 100 mM d-Gal was also significantly lower compared to the control. Thus, pre-treatment with Q did not restore the GSH/GSSG ratio. Q alone (10 µM) did not significantly affect the GSH/GSSG ratio.

## 3. Discussion

Over time, every cell loses its effectiveness for defending itself against reactive oxygen species (ROS) and other free radical species, which leads to progressive cellular oxidation. Blood plays a special role in the aging mechanism, as it is not only a supplier of oxygen and nutrients for tissues, but it also removes metabolic waste and oxidative species. In a cell-based model of accelerated aging, exposure to d-Gal affected lipid chains and proteins in the erythrocyte membrane, which was revealed by an increase in lipid peroxidation levels and a decrease in sulfhydryl group content, as well as an increased hemoglobin glycation [29]. Several studies suggest that plant polyphenols can be promising agents for pharmacological interventions against specific age-associated diseases through a variety of beneficial biological mechanisms [24]. Therefore, in the present study, Q was used to investigate the potential protective role of polyphenols against OS and glycation associated with aging in erythrocytes. In particular, Q has been shown to be an excellent antioxidant in vitro [52] and a protective compound [53]. Cherrak and co-authors [54] reported that Q exhibited strong antioxidant properties toward heavy metals and contrasted their hemolytic activity and ability to increase the lipid peroxidation of membrane erythrocytes. Other authors described Q as a good natural product able to increase membrane fluidity during hypercholesterolemia [55]. Because the mechanism of action of Q is still unclear, in line with previous studies on natural antioxidants, such as curcumin and melatonin, in erythrocytes [51,56], our study was conducted to explore the possible protective effect of Q in an erythrocyte model of aging induced by d-Gal.

The first step of this work was to assess the erythrocyte tolerance to Q. Therefore, a preliminary screening by TBARS level measurements was conducted. This test let us exclude any damage in terms of lipid peroxidation that could potentially be caused by Q alone. At the same time, the first evidence of the beneficial effect of a wide range of Q concentrations (from 10 µM to 1 mM) has been obtained, in agreement with other authors [57,58]. In particular, Q has been effective in preventing lipid peroxidation induced by 10 mM H_2_O_2_ (Figure 2), which was associated with OS [59]. Based on this evidence, and considering that the focus of the present investigation is related to aging, which is reportedly associated with OS events [60], 10 µM Q was chosen as the lowest effective concentration and was tested in a model of aging in human erythrocytes.

One of the most interesting and still unclear implications of aging is its impact on membrane transport systems. In this context, different studies reported that the process of aging may affect the bicarbonate/chloride exchanger (B3p) in the erythrocyte membrane. This membrane transport system is involved in crucial physiological functions such as gas exchange and represents a major site of anchoring of the cytoskeleton to the erythrocyte membrane [61]. Natural aging-dependent oxidation of the membrane B3p increases the affinity for circulating anti-B3p antibodies, thus inducing modifications in the erythrocyte structure, including decreased deformability and increased sphericity. These immunocomplexes, in turn, are recognized by macrophages, which actively clear erythrocytes from the blood circulation [62,63]. Recently, our group [29] has reported that erythrocytes exposed to d-Gal (25, 35, 50, and 100 mM for 24 h) are characterized by both an increase in OS and functional alterations on the B3p reflected by an accelerated rate constant for SO_4_^2−^ uptake. Among the experimental models of aging, long-term d-Gal exposure is the most similar to natural aging. d-Galactose is a reducing sugar, whose abnormally increased levels can be converted into d-Gal exodialdose and hydroperoxide by the galactose oxidase enzyme, thus resulting in the generation of ROS and OS. At the same time, d-Gal can initiate non-enzymatic glycation reactions to form AGEs. Among the many cellular models used to investigate the biochemical alterations during aging, as well as the impact of OS, erythrocytes are a good model [8,33,64]. In this regard, in the present study, we hypothesized that Q could exert a beneficial action during aging that could be revealed by protection of the B3p function. Hence, the second step of the present work was to measure the SO_4_^2^^−^ uptake after a 24 h treatment with d-Gal (100 mM) with a validated method to assay anion-exchange capability through the B3p. Under these experimental conditions, the rate constant for SO_4_^2^^−^ uptake was accelerated and, in parallel, the amount of internalized SO_4_^2^^−^ was significantly increased (Figure 3, Table 1). This was in agreement to former findings [29]. One-hour pre-treatment with 10 µM Q completely restored the rate constant for the SO_4_^2^^−^ uptake, which, on one hand, demonstrated a protective effect of Q on the B3p function and, on the other hand, led us to further explore the antioxidant effect of Q in erythrocytes [65].

Although Q displays antioxidant effects in several cells and tissues, some studies demonstrated that Q can convert to reactive oxidation products, namely o-semiquinone and/or o-quinone, which could react with thiols, causing a loss of protein function, as well as cytotoxic effects [66]. The occurrence of possible anti- rather than pro-oxidative effects of Q could therefore depend on its dose, the time of exposure, and the cellular redox state [67]. In this context, to better explain the mechanisms through which the anion-exchange capability through B3p is affected by Q, two specific parameters correlated to B3p function [8,29], i.e., lipid peroxidation and protein oxidation, have been considered. Exposure to 100 mM d-Gal produced a significant oxidation of lipids (Figure 4), in line with what was already proved [29,68]. This damage was effectively prevented in the erythrocytes treated with 10 µM Q, during human aging. With regard to proteins, d-Gal exposure induced a significant decrease in the total sulfhydryl groups, which was only partially restored by the Q treatment (Figure 5). These findings suggest that the efficiency of Q in preventing age-related damage in this experimental model is differently achieved at the plasma membrane level. In particular, the protective effect of Q, at least in the dose of 10 µM, seems more targeted on membrane lipids rather than proteins. Our previous studies suggested that alteration of the SO_4_^2−^ transport kinetics could be linked to mechanisms other than oxidative stress, for example, glycated Hb formation [69]. In this regard, our attention has been addressed to a possible anti-glycant effect of Q, which is still poorly explored in erythrocytes. Therefore, the glycated hemoglobin content (%A1c) was measured. The results confirmed that the d-Gal-treated erythrocytes (100 mM) showed an increase in glycated hemoglobin (Figure 6), which was fully prevented by pre-treatment with 10 µM Q. Thus, these results confirm that post-translational changes of intracellular proteins, including hemoglobin, might affect B3p function during aging and further support a significant anti-glycation effect of Q on hemoglobin.

At last, our focus has been directed to the endogenous antioxidant system; hence, the GSH/GSSG ratio was evaluated. The results confirmed that 100 mM d-Gal treatment reduced the GSH/GSSG ratio. However, pre-incubation of erythrocytes with 10 µM Q had minimal beneficial effect on the redox balance (Figure 7). The decline in antioxidant capacity emphasizes the importance of the role of OS in human aging [70,71]. Moreover, it is known that during its antioxidative activities, Q yields the oxidation product quercetin–quinone, denoted as QQ [52]. Indeed, QQ is very reactive toward thiols and can instantaneously form an adduct with GSH, the most abundant endogenous thiol. The adduct is rapidly dissociated into GSH and QQ with a half-life of 2 min [72]. It has been shown that, as long as the GSH concentration is high, it will offer protection against QQ by trapping it as GSQ [72]. However, when the GSH concentration is low, the dissociated QQ will react with other thiol groups, such as protein sulfhydryl groups. The binding of QQ to these other thiols could lead to toxic effects, increase OS, and alter antioxidant enzymes that contain -SH groups. On this basis, our study showed that the GSH/GSSG ratio is low in our model of aging. We hypothesize that the formation of a QQ adduct might have led to oxidative damage on membrane proteins, possibly explaining the partial restoration of total sulfhydryl groups obtained with Q (Figure 5) compared to the full protective effect on lipids (Figure 4). QQ-induced toxicity has been shown in various in vitro studies and has been recently defined as the Q paradox [73]. Therefore, we confirmed that Q might not enhance the endogenous antioxidant system (GSH-GSSH) against oxidation induced by natural aging.

## 4. Materials and Methods

### 4.1. Solution and Chemical Products

All chemicals were purchased from Sigma (Milan, Italy). Both 3,3,4,5,7-pentahydroxyflavone (Q) (10 mM) and 4,4′-diisothiocyanatostilbene-2,2′-disulfonate (DIDS) (10 mM) stock solutions were prepared in dimethyl sulfoxide (DMSO). D-Galactose stock solution (1 mM) was prepared in distilled water. Both N-ethylmaleimide (NEM) (310 mM) and 5,5-dithio-bis-(2-nitrobenzoic acid) (DTNB) (50 mM) stock solutions were prepared in ethanol. H_2_O_2_ was diluted in distilled water from a 30% *w*/*w* stock solution. Both ethanol and DMSO never exceeded 0.001% *v*/*v* in the experimental solutions and were previously tested on erythrocytes to exclude hemolysis.

### 4.2. Erythrocyte Preparation

**Figure 8:**
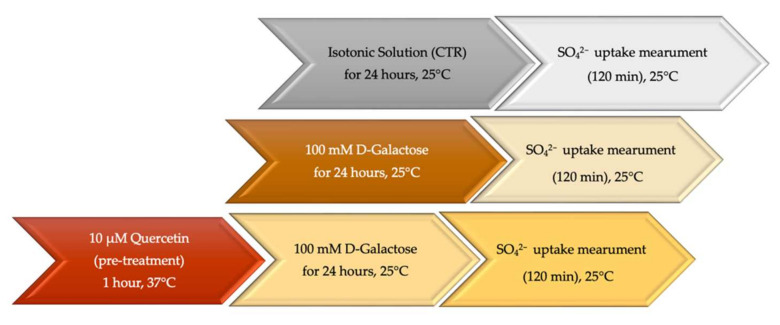
Time course of experimental procedures before SO_4_^2−^ uptake measurement.

Whole human blood from male healthy volunteers (age 25–45 years) was collected in test tubes containing ethylenediaminetetraacetic acid (EDTA). Plasma concentration of glycated hemoglobin (A1c) was less than 5%. Erythrocytes were washed in isotonic solution (composition in mM: NaCl 150, 4-(2-hydroxyethyl)-1-piperazineethanesulfonic acid (HEPES) 5, Glucose 5, pH 7.4, osmotic pressure 300 mOsm/kgH_2_O) and centrifuged thrice (Neya 16R, 1200× *g*, 5 min) to remove plasma and buffy coat. Erythrocytes were then suspended at different hematocrit in isotonic solution and addressed to different treatments according to the experimental design reported in Figure 8.

### 4.3. Thiobarbituric Acid Reactive Substances (TBARS) Level Measurements

TBARS levels were measured as described by Mendanha and collaborators [74], with few modifications. TBARS derive from the reaction between thiobarbituric acid (TBA) and malondialdehyde (MDA), which is the end-product of lipid peroxidation [40]. Erythrocytes were suspended at 20% hematocrit and pre-incubated for 1 h with different Q concentrations (10, 50, 1000 µM) at 37 °C. Successively, samples were incubated with 100 mM d-Gal for 24 h. Then, samples were centrifuged (Neya 16R, 1200× *g*, 5 min) and suspended in isotonic solution. A total of 1.5 mL of erythrocytes was treated with 10% (*w*/*v*) trichloroacetic acid (TCA) and centrifuged (Neya 16R, 3000× *g*, 10 min). A total of 1 mL of TBA (1% in hot distilled water) was added to the supernatant and the mixture was incubated at 95 °C for 30 min. At last, TBARS levels were obtained by subtracting 20% of the absorbance at 453 nm from the absorbance at 532 nm (Onda Spectrophotometer, UV-21). Results are indicated as µM TBARS levels (1.56 × 10^5^ M^−1^ cm^−1^ molar extinction coefficient).

### 4.4. SO_4_^2−^ Uptake Measurement

#### 4.4.1. Control Condition

SO_4_^2−^ uptake measurement was used to evaluate the anion exchange through B3p, as described elsewhere [48,75,76,77]. Briefly, after washing, erythrocytes were suspended to 3% hematocrit in 35 mL SO_4_^2−^ medium (composition in mM: Na_2_SO_4_ 118, HEPES 10, glucose 5, pH 7.4, osmotic pressure 300 mOsm/kgH_2_O) and incubated at 25 °C. After 5, 10, 15, 30, 45, 60, 90, and 120 min, DIDS (10 μM), which is an inhibitor of B3p activity [78], was added to 5 mL sample aliquots, which were kept on ice. Subsequently, samples were washed three times in cold isotonic solution and centrifuged (Neya 16R, 4 °C, 1200× *g*, 5 min) to eliminate SO_4_^2^^−^ from the external medium. Distilled water (1 mL) was added to induce osmotic lysis of erythrocytes, and perchloric acid (4% *v*/*v*) was used to precipitate proteins.

After centrifugation (Neya 16R, 4 °C, 2500× *g*, 10 min), the supernatant containing SO_4_^2^^−^ trapped by erythrocytes was directed to the turbidimetric analysis. A total of 500 μL supernatant from each sample was sequentially mixed to 500 μL glycerol diluted in distilled water (1:1), 1 mL 4 M NaCl, and 500 μL 1.24 M BaCl_2_·2H_2_O. Finally, the absorbance of each sample was measured at 425 nm (Onda Spectrophotometer, UV-21). By means of a calibrated standard curve previously obtained by precipitating known SO_4_^2^^−^ concentrations, the absorbance was converted to [SO_4_^2^^−^] L cells × 10^−2^ to calculate the rate constant of SO_4_^2^^−^ uptake (min^−1^), which is derived from the following equation: C_t_ = C_∞_ (1 − e^−rt^) + C_0_, where C_t_, C_∞_, and C_0_ indicate the intracellular SO_4_^2^^−^ concentrations measured at time t, ∞, and 0, respectively, where e represents the Neper number (2.7182818), r indicates the rate constant accounting for the process velocity, and t is the specific time at which the SO_4_^2−^ concentration was measured. The rate constant is the inverse of the time needed to reach ~63% of total SO_4_^2^^−^ intracellular concentration [75], and [SO_4_^2^^−^] L cells × 10^−2^ reported in figures represents SO_4_^2^^−^ micromolar concentration internalized by 5 mL erythrocytes suspended at 3% hematocrit.

#### 4.4.2. Experimental Conditions

After 1 h incubation with or without Q (10 µM) at 37 °C, erythrocytes (3% hematocrit) were exposed to d-Gal (100 mM) for 24 h at 25 °C. Successively, samples were centrifuged (Neya 16R, 4 °C, 1200× *g*, 5 min) to replace the supernatant with SO_4_^2−^ medium. The rate constant of SO_4_^2−^ uptake was then determined as described for the control condition.

### 4.5. Total Sulfhydryl Group (-SH) Content Measurement

Measurement of total -SH groups was carried out according to the method of Aksenov and Markesbery [79], with few modifications. In short, after incubation in **d**-Gal-containing solutions with or without Q (35% hematocrit), erythrocytes were centrifuged (Neya 16R, 1200× *g*, 5 min) and 100 µL of red cells hemolyzed with 1 mL of distilled water. A 50 μL aliquot of this mixture was added to 1 mL of phosphate-buffered saline (PBS; pH 7.4) containing EDTA (1 mM). A total of 30 μL of 5,5′-Dithiobis (2-nitrobenzoic acid) (DTNB, 10 mM) was added to initiate the reaction, and the samples were incubated for 30 min at 25 °C protected from light. Control samples, without proteins or DTNB, were processed concurrently. After incubation, samples absorbance was measured at 412 nm (Onda spectrophotometer, UV-21), and 3-thio-2-nitro-benzoic acid (TNB) levels were detected after subtraction of blank absorbance (samples containing only DTNB). To achieve full oxidation of -SH groups, an aliquot of erythrocytes (positive control) was incubated with NEM (2 mM) for 1 h at 25 °C [43,56]. Data were normalized to protein content and results reported as µM TNB/mg protein.

### 4.6. Measurement of Glycated Hemoglobin (%A1c)

The glycated hemoglobin content (%A1c) was determined with the A1c liquidirect reagent as previously described by Sompong and collaborators [80], with slight modifications. Briefly, after incubation with **d**-Gal, with or without pre-incubation with Q, samples were lysed in hypotonic buffer and then incubated with latex reagent at 37 °C for 5 min. The absorbance of samples was measured at 610 nm (Eppendorf, BioPhotometer Plus), and the A1c content was calculated from a standard curve constructed by using known A1c concentrations and expressed in %.

### 4.7. Measurement of GSH/GSSG Ratio

GSH/GSSG ratio was determined according to Giustarini and collaborators [81], with few modifications. This assay is based on the reduced glutathione (GSH) recycling method. GSH conjugation with the Ellman’s reagent DTNB produces the chromophore 3-thio-2-nitro-benzoic acid (TNB) and the mixed disulphide GS-TNB. GS-TNB is then reduced by glutathione reductase (GR) in the presence of reduced nicotinamide adenine dinucleotide phosphate (NADPH), forming GSH and TNB. The latter can be spectrophotometrically detected at 412 nm. After treatment with d-Gal (100 mM), with or without pre-exposure to Q (10 µM), the content of GSH was measured as described above. The content of GSSG was calculated by the following formula: 1/2 GSSG = GSH_total_ − GSH_reduced_. Results are expressed as a GSH/GSSG ratio [69].

### 4.8. Experimental Data and Statistics

All data are expressed as arithmetic means ± standard errors of the mean. For statistical analysis and graphics, GraphPad Prism (version 8.0, GraphPad Software, San Diego, CA, USA) and Excel (Version 2019, Microsoft, Redmond, WA, USA) software were used. Data normality was verified with the D’Agostino and Pearson Omnibus normality test. Significant differences between mean values were determined by one-way analysis of variance (ANOVA), followed by Bonferroni’s multiple comparison post hoc test, or ANOVA with Dunnet’s post-test, as appropriate. Statistically significant differences were assumed at *p* < 0.05; (*n*) corresponds to the number of separate experiments.

## 5. Conclusions

In the present study, we explored for the first time the impact of Q on the B3p anion-exchange capability during erythrocyte aging, along with glycation and oxidative events. Exposure of erythrocytes to 100 mM D-Gal for 24 h was chosen as an experimental model of early aging. In this model, the anion-exchange capability via the B3p exhibited an accelerated rate constant for SO_4_^2−^ uptake, which was associated with glycated hemoglobin formation, a decrease in the endogenous antioxidant system, and increased OS at both the lipid and protein levels. Quercetin acted on each of these parameters to different extents. We conclude that 10 µM Q (i) protected B3p function in a D-Gal-induced erythrocyte aging model; (ii) effectively prevented oxidative damage on membrane lipids; (iii) partially protected cellular proteins from oxidation, putatively due to the Q paradox; (iv) had an anti-glycant effect in preventing A1c formation; and (v) had no beneficial effect on the endogenous GSH/GSSH balance. In this light, the assessment of B3p function appears to be a sensitive tool to monitor erythrocytes homeostasis also in an experimental model of aging and identifies the B3p as a potential target of antioxidant treatments to counteract aging-related disturbances. Further studies aimed at defining the intracellular signaling underlying Q action are needed to better ascertain whether this flavonoid may effectively be utilized in pharmacological strategies against oxidative stress during aging.

## Figures and Tables

**Table 1:** Rate constant of SO_4_^2−^ uptake and amount of SO_4_^2−^ trapped in untreated (control) erythrocytes and erythrocytes treated as indicated. Data are presented as means ± S.E.M. from separate (n) experiments, where ns, not statistically significant versus untreated (control); ***, *p* < 0.001 versus control, ^§§§^, *p* < 0.001 versus 100 mM d-Gal-treated erythrocytes, one-way ANOVA followed by Bonferroni’s multiple comparison post hoc test.

Experimental Conditions	Rate Constant (min^−1^)	Time (min)	n	SO_4_^2−^ Amount Trapped after 45 min of Incubation in SO_4_^2−^ Medium [SO_4_^2−^] L Cells × 10^−2^
Untreated (control)	0.064 ± 0.001	15.47	10	379.60 ± 18.63
10 µM Q	0.063 ± 0.002 ^ns^	15.64	10	388.35 ± 19.90 ^ns^
100 mM d-Gal	0.106 ± 0.001 ***	9.36	7	524.35 ± 16.50 ***
10 µM Q + 100 mM d-Gal	0.080 ± 0.001 ^§§§^	12.32	5	349.97 ± 11.15 ^§§§^
10 µM DIDS	0.016 ± 0.001 ***	62.50	5	5.49 ± 3.50 ***

## Data Availability

The data that support the findings of this study are available from the corresponding author upon reasonable request.
